# Cancer, cardiovascular disease and diabetes mortality among women with a history of endometrial cancer

**DOI:** 10.1038/sj.bjc.6603761

**Published:** 2007-04-24

**Authors:** S H Wild, J R Bryden, R J Lee, J L Bishop, A R Finlayson, C D Byrne, D H Brewster

**Affiliations:** 1 Public Health Sciences, University of Edinburgh, Scotland, UK; 2 Information Services, NHS National Services Scotland, UK; 3 Department of Endocrinology, University of Southampton, UK

**Keywords:** endometrial cancer, mortality, breast cancer, ovarian cancer, cardiovascular disease, diabetes

## Abstract

Among 7182 women with endometrial cancer in Scotland, standardised mortality ratios (and 95% confidence intervals (CI)) were 6.38 (5.74–7.15) for all cancers and 1.10 (1.00–1.22) for circulatory diseases as underlying cause of death and 2.81 (2.19–3.70) for diabetes as underlying/contributory cause of death.

Endometrial cancer (malignant neoplasm of the corpus uteri) is one of the five most common cancers among women and develops in approximately 400 women in Scotland (ISD Scotland, 2004), 40 000 women in the United States (National Cancer Institute, 2006) and 77 000 women across Europe each year (International Agency for Research on Cancer, 2005). Age-standardised incidence of endometrial cancer in Scotland has increased over the last decade and mortality rates appear stable (ISD Scotland, 2004). Compared with many other cancers, the survival after a diagnosis of endometrial cancer is high, with survival relative to the age-matched general population of women of approximately 70%, five years after diagnosis (Harris *et al*, 1998; Quinn *et al*, 2001). It is therefore important to consider the role of other causes of morbidity and mortality among women with endometrial cancer.

Endometrial cancer is associated with factors that increase (including obesity, diabetes and hypertension) (Shoff and Newcomb, 1998; Parazzini *et al*, 1999; Anderson *et al*, 2001; Xu *et al*, 2005, 2006; Trentham-Dietz *et al*, 2006) and decrease (including higher socio-economic status and not smoking) (Kelsey *et al*, 1982) risk of cardiovascular disease. In addition, there is an association with polycystic ovary syndrome (Hardiman *et al*, 2003), the effect on cardiovascular disease of which is unclear. It is plausible that women who survive endometrial cancer may be at high risk of cardiovascular disease and could benefit from effective approaches to primary and secondary prevention.

In Scotland, linkage between several centrally held datasets including cancer registries and death records is performed using probability matching (Kendrick and Clarke, 1993), which uses all available identifying information, including name, date of birth, postal code and patient reference number, to link records belonging to the same person.

We examined the causes of death among women with endometrial cancer and compared mortality from all causes, all cancers and selected major cancers, all diseases of the circulatory system (specifically ischaemic heart disease and stroke) and diabetes between women with endometrial cancer and women in the general population of Scotland.

## MATERIALS AND METHODS

The Information Services Division of National Health Service National Services Scotland identified a retrospective cohort of women in Scotland who received a registration of malignant neoplasm of the corpus uteri (ninth and tenth revisions of the International Classification of Diseases (ICD-9 and ICD-10) codes 182 and C54) between 1981 and 2000 and provided anonymised data linked to mortality records for cohort members.

Cause of death in Scotland was coded using ICD-9 codes for deaths between 1981 and 1999 and ICD-10 codes for deaths occurring after 1999. An area-based measures of deprivation category was used as a marker for socio-economic status, which is a potentially important confounding factor. The Carstairs Index is derived from four census indicators: low social class, lack of car ownership, overcrowding and male unemployment for postal sectors derived from 1981 and 1991 census data (Carstairs and Morris, 1991).

Estimates of the numbers of women in Scotland by age group (in 5-year groups from 30 to 34 and 85+), calendar period (1981–1985, 1986–1990, 1991–1996, 1997–2002) and deprivation category were derived from the Registrar General for Scotland mid-year population estimates and 1981 and 1991 census population estimates. The number of deaths for the general population of women in Scotland by 5-year age group, calendar period and deprivation category were available for all causes combined and for selected underlying causes of death: all cancers (ICD-9: 140–208, ICD-10: C0-C97), breast cancer (ICD-9: 174, ICD-10: C50), ovarian cancer (ICD-9: 183, ICD-10: C56), colon cancer (ICD-9: 153, ICD-10: C18), all diseases of the circulatory system (ICD-9: 390–459, ICD-10: I00-I99), ischaemic heart disease (ICD-9: 410–414, ICD-10: I20-I25), cerebrovascular disease (ICD-9: 430–438, ICD-10: I60-I69, G45). Diabetes (ICD-9: 250, ICD-10: E10-E14) was considered as either an underlying or contributory cause of death.

For each cohort member, date of endometrial cancer diagnosis was grouped into calendar periods of 1981–1985, 1986–1990, 1991–1995 and 1996–2000. Length of follow-up was calculated in days either between diagnosis and death or between diagnosis and the end of 2002 for survivors. Calendar periods at death were grouped in the following categories: 1981–1985, 1986–1990, 1991–1996 and 1997–2002. The analysis was based on women of 30 years and older at diagnosis who had complete data available on age, deprivation category and date of diagnosis.

Standardised mortality ratios (SMRs) for all combined and selected causes of death were derived for the endometrial cancer cohort using indirect standardisation. Age group, calendar period and deprivation category-specific mortality rates for the whole population of women in Scotland over 29 years of age (approximately 1.7 million women) were used as the standard. CIs were estimated using the Fieller method, which takes account of potential errors in expected death rates (Silcocks, 1994).

## RESULTS

The cohort included 16 women below 30 years of age and four women with missing data for deprivation category and these 20 women were excluded, leaving 7182 women in the analysis. The mean and median age at diagnosis of endometrial cancer was 65 years. Median time between diagnosis of endometrial cancer and death or the end of the follow-up period at 31 December 2002 for the cohort was 5.0 years (inter-quartile range: 1.8–10.5 years).

Approximately half of the cohort (3615 women) died during the follow-up period and five year absolute survival was 62%. The single largest cause of death was endometrial cancer with 1529 (42%) death certificates giving endometrial cancer (ICD-9 and ICD-10 codes 182 and C54) as the underlying cause of death. There were another 210 (5.8%) death certificates that gave ICD-9 and ICD-10 codes 179 and C55 (uterus, part unspecified) for the underlying cause of death. A further 384 death certificates (11%) included endometrial cancer (ICD-9 and ICD-10 codes 182 and C54) as a contributory cause of death.

Breast cancer, ovarian cancer, ischaemic heart disease and cerebrovascular disease were identified as the underlying cause of death for 114 (3.2%), 87(2.4%), 394 (11%), 238 (6.6%) deaths, respectively. Diabetes was reported as either underlying or contributory cause of death on 220 death certificates (6.1%). Diabetes was given as the underlying cause of death on only 32 death certificates. The most common underlying causes of death when diabetes was given as a contributory cause were ischaemic heart disease (52 deaths), endometrial cancer (29 deaths) and cerebrovascular disease (24 deaths). Table 1 shows SMRs for all causes, all cancers, endometrial, breast and ovarian cancer, all circulatory diseases, ischaemic heart disease, cerebrovascular disease and diabetes among the cohort of women with endometrial cancer.

## DISCUSSION

In this first cohort study of mortality in Britain among women with endometrial cancer we found that over 42% of death certificates included endometrial cancer as the underlying cause of death. We found that in the cohort, ovarian cancer mortality was over four times higher and all-cause, breast cancer and diabetes-related mortality was approximately twice as high as that of the general female population, after allowing for potential differences in distribution of age, calendar period and deprivation category between groups. In contrast, mortality from ischaemic heart disease and cerebrovascular disease were similar in the cohort and the general female population.

A similar proportion of deaths ascribed to endometrial cancer was also reported by a smaller study in Iowa in which 39 out of 93 death certificates for women with a history of endometrial cancer gave endometrial cancer as the underlying cause of death (Folsom *et al*, 2004). The high breast cancer mortality in the endometrial cancer cohort reflects shared risk factors (including low parity, late menopause, high socio-economic status and obesity) but perhaps also the increased endometrial cancer risk associated with tamoxifen treatment for breast cancer (Bergman *et al*, 2000). Tamoxifen treatment appears to be associated with a reduced risk of cardiovascular disease (Swerdlow and Jones, 2005). We were unable to determine in our study the temporal relationship between endometrial and breast cancers. Higher ovarian cancer mortality in the cohort may again be related to shared risk factors as for breast cancer, but could also be potentially related to oestrogen production by some ovarian cancers.

Recording of diabetes on death certificates is known to be incomplete (Thomason *et al*, 2005). Under-reporting of diabetes on death certificates appears to be even more marked for people who died of cancer than among those with other recorded causes of death (McEwen *et al*, 2006). This would suggest a bias that may underestimate excess diabetes-related mortality among women with endometrial cancer.

The relative risk of coronary heart disease among women with diabetes is approximately 2.5-fold that of women without diabetes (Lee *et al*, 2000). The presumed higher prevalence of diabetes among women with endometrial cancer could therefore be expected to be associated with an increase in cardiovascular disease mortality. A long-term follow-up study of women with polycystic ovary syndrome also reported no excess of cardiovascular disease mortality, despite an excess of prevalence of and mortality from diabetes (Wild *et al*, 2000). The absence of excess cardiovascular disease mortality in the cohort could potentially be explained by a combination of endometrial and breast cancer acting as competing causes of death, residual confounding, non-random misclassification bias or possibly a protective effect of exposure to unopposed oestrogen against cardiovascular disease. A prospective cohort study is required to establish the role of these factors.

The use of routine data means that the potential confounding effects of only age, calendar period and deprivation category could be addressed in this study. We were unable to consider the effects of other potential confounding factors including parity, body mass index, fat distribution, smoking habit, presence and treatment for hypertension or dyslipidaemia and use of hormone replacement therapy, aspirin, anti-hypertensives, lipid-lowering drugs or tamoxifen.

There is the potential for errors in record linkage, but this is unlikely to have a marked effect as the linkage process is estimated to be 98% accurate (Harley and Jones, 1999). The codes and the rules for coding the underlying cause of death changed with the introduction of ICD-10 codes for mortality data in 2000 and we have assumed that any effect of this would be similar for women with and without endometrial cancer.

In conclusion, we have shown that endometrial cancer is associated with an approximately fourfold increase in ovarian cancer mortality and a twofold increase in mortality from breast cancer and diabetes, and that cardiovascular disease mortality among women with a history of endometrial cancer is similar to that observed in the general population. The findings suggest that women with endometrial cancer should receive similar advice on lifestyle and pharmacological treatment for primary and secondary prevention of cardiovascular disease to women in the general population. The high diabetes prevalence and mortality among women with endometrial cancer suggests that weight control and regular physical activity are particularly important for this group of women. Further research is required to establish the temporal relationships between endometrial cancer, ovarian cancer, breast cancer and diabetes and to establish appropriate approaches to screening for and management of each condition among women with one or more of the other conditions.

## Figures and Tables

**Table 1:** Numbers of deaths, mortality ratios (SMR) and 95% CI for selected causes of death between 1981 and 2002 among women with endometrial cancer diagnosed between 1981 and 2000 in Scotland, standardised for age, deprivation category and calendar period

**Cause of death (ICD codes)**	**Deaths**	**SMR**	**95% CI**
All causes	3615	2.34	2.21–2.49
All cancers (ICD-9: 140–208, ICD-10: C0–C97)	2357	6.38	5.74–7.15
Endometrial cancer (ICD-9: 182, ICD-10: C54)	1529	277	150–1682
Breast cancer (ICD-9: 174, ICD-10: C50)	114	2.05	1.51–2.90
Ovarian cancer (ICD-9: 183, ICD-10: C56)	87	4.26	2.77–7.74
All circulatory disease (ICD-9: 390–459, ICD-10: I00–I99)	795	1.10	1.00–1.22
Ischaemic heart disease (ICD-9: 410–414, ICD-10: I20–I25)	394	1.10	0.96–1.27
Cerebrovascular disease (ICD-9: 430–438, ICD-10: I60–I69, G45)	238	1.02	0.85–1.22
Diabetes (underlying or contributory cause) (ICD-9: 250, ICD-10: E10–E14)	220	2.81	2.19–3.70

Abbreviations: CI, confidence intervals; SMR, standardized mortality ratios.
